# Development and validation of a risk prediction model for frailty in patients with diabetes

**DOI:** 10.1186/s12877-023-03823-3

**Published:** 2023-03-27

**Authors:** Fan Bu, Xiao-hui Deng, Na-ni Zhan, Hongtao Cheng, Zi-lin Wang, Li Tang, Yu Zhao, Qi-yuan Lyu

**Affiliations:** School of Nursing, Jinan University, No. 601, West Huangpu Avenue, Guangzhou, People’s Republic of China

**Keywords:** Predictive model, Frailty, Diabetics, Diabetic patients

## Abstract

**Background:**

Frailty is the third most common complication of diabetes after macrovascular and microvascular complications. The aim of this study was to develop a validated risk prediction model for frailty in patients with diabetes.

**Methods:**

The research used data from the China Health and Retirement Longitudinal Study (CHARLS), a dataset representative of the Chinese population. Twenty-five indicators, including socio-demographic variables, behavioral factors, health status, and mental health parameters, were analyzed in this study. The study cohort was randomly divided into a training set and a validation set at a ratio of 70 to 30%. LASSO regression analysis was used to screen the variables for the best predictors of the model based on a 10-fold cross-validation. The logistic regression model was applied to explore the associated factors of frailty in patients with diabetes. A nomogram was constructed to develop the prediction model. Calibration curves were applied to evaluate the accuracy of the nomogram model. The area under the receiver operating characteristic curve and decision curve analysis were conducted to assess predictive performance.

**Results:**

One thousand four hundred thirty-six patients with diabetes from the CHARLS database collected in 2013 (*n* = 793) and 2015 (*n* = 643) were included in the final analysis. A total of 145 (10.9%) had frailty symptoms. Multivariate logistic regression analysis showed that marital status, activities of daily living, waist circumference, cognitive function, grip strength, social activity, and depression as predictors of frailty in people with diabetes. These factors were used to construct the nomogram model, which showed good concordance and accuracy. The AUC values of the predictive model and the internal validation set were 0.912 (95%CI 0.887–0.937) and 0.881 (95% CI 0.829–0.934). Hosmer–Lemeshow test values were *P* = 0.824 and *P* = 0.608 (both > 0.05). Calibration curves showed significant agreement between the nomogram model and actual observations. ROC and DCA indicated that the nomogram had a good predictive performance.

**Conclusions:**

Comprehensive nomogram constructed in this study was a promising and convenient tool to evaluate the risk of frailty in patients with diabetes, and contributed clinicians to screening the high-risk population.

**Supplementary Information:**

The online version contains supplementary material available at 10.1186/s12877-023-03823-3.

## Background

There are more than 140 million people with diabetes in China, which is the highest in the world. The number is expected to reach 174 million by 2045 [1]. Frailty has become the third major complication of diabetes after macrovascular and microvascular complications [2], and it is an independent risk factor for death and disability in people with diabetes [3, 4]. Frailty, defined as loss of fitness associated with age and disease, is a fragile state with poor homeostasis resolution after stress and a consequence of the decline of multiple physiological systems [5].

The prevalence of frailty in people with diabetes is up to 48%, and the probability of developing frailty is three to five times higher than that in people without diabetes [6]. Frailty not only increases the risk of adverse events, such as fractures, falls, disability, and hospitalization in people with diabetes [7, 8], but also increases medical and health expenditure [9]. Additionally, diabetics with frailty due to diabetes have a higher mortality rate than those without frailty [10]. An increase in frailty score by one unit results in a 93% increased risk of long-term mortality in patients with diabetes, and people with both diabetes and frailty have a 2.62 times higher risk of complication than non-frail diabetic patients [4].

The development of frailty in patients with diabetes is a consequence of the combined effects of multiple factors. A previous study indicated that gender, age, and socioeconomic status are all independently associated with the development of frailty [7]. Another study further revealed that hyperglycemia, hypoglycemia, low hemoglobin A1c (HbA1c), insulin resistance, cardiovascular disease, low physical activity, and malnutrition increase the risk of frailty among patients with diabetes [11]. Yakabe and Ogawa found that chronic conditions, such as visual impairment, diabetic complications, comorbidities, and depression, may also contribute to frailty in patients with diabetes [12].

The development of frailty is dynamic and reversible [13]. Early screening for the high-risk population is important for early intervention to delay the onset and progression of frailty. Risk prediction models are a tool that can be used to assess the risk of frailty occurring among patients with diabetes. Most previous studies have focused on investigating the status of frailty and its influencing factors, while few have attempted to develop risk prediction models to screen subjects at high risk of frailty. For example, Dong and associates [14] developed and validated a frailty prediction model in community-living older adults. The study indicated that age, marital status, physical exercise, baseline frailty state, and diabetes were independently associated with frailty. Li and colleagues [15] constructed a dynamic simulation model to predict frailty and concluded that older age, working in a professional or technical role before 60 years old, poor economic status, and poor oral hygiene were independent risk factors for frailty.

Nevertheless, the present models were based only on healthy populations, and no predictive model for frailty in patients with diabetes has been reported. This study aimed to identify factors associated with frailty and incorporate them into a nomogram constructed based on a model for predicting frailty in patients with diabetes.

## Methods

### Study design

We used data from the China Health and Retirement Longitudinal Study (CHARLS), which was publicly available at http://charls.pku.edu.cn. The project was approved by the Biomedical Ethics Committee of Peking University (Beijing, China). The data is of high quality and has the nature of a large sample, thus providing real and effective data support for the analysis of this paper. Data from CHARLS 2013 and 2015 were selected for analysis in this study. After excluding participants with missing data, one thousand four hundred thirty-six patients were included in the analyses. Our research has been performed in accordance with the Declaration of Helsinki. The original CHARLS was approved by the Ethical Review Committee of Peking University (IRB00001052–11,015), and all participants signed the informed consent at the time of participation.

### Data collection

#### Frailty

The definition of frailty was originally proposed by Fried et al. [16] and includes unintentional weight loss, self-reported exhaustion, weakness, slow walking speed, and low physical activity. Based on the above criteria, and combined with the information available from the CHARLS database and previous research, modified criteria have emerged and the previous study has justified that the criteria are equally valid for frailty [17]. This study refers to the diagnostic criteria of frailty from the previous study.

It includes exhaustion, weakness, low physical activity, weight loss, and slowness. In this study, frailty was treated as a binary outcome indicator and the specific assessment is as follows:

- 1. Weakness was measured using the self-reported item “having difficulty in lifting or carrying weights over 5 kg” [18].
- 2. Slowness was considered present if a participant had difficulty walking 100 m or climbing several flights of stairs without resting, which was similar to that used in previous studies [18].
- 3. Exhaustion was present if the participant answered “Most or all of the time” or “Occasionally or a moderate amount of the time”, in response to either of the Chinese version of the Center for Epidemiologic Studies-Depression scale (CES-D) questions: “I felt everything I did was an effort during last week” or “I could not get going during last week.” This variable was constructed identically to the originally proposed by Fried et al. [16].
- 4. Low physical activity was considered to be present if the participants did not undertake physical activity or walk at least 10 min at a time during a usual week. This variable is different from that proposed by Fried et al. [16], but a similar study has previously used this variable to determine frailty [19].
- 5. Weight loss was defined as unintentional loss of 5 or more kg in the last year or current body mass index (BMI) ≤ 18.5 kg/m^2^ [17]. It has been proved that weight loss was a better indicator of frailty than body mass index and energy intake [20].

Frailty was defined as the presence of three or more of the above five components.

#### Socio-demographic factors

Socio-demographic factors included age, gender, education level, marital status, permanent address, insurance, and financial support. Gender was defined as either male or female. Education level was categorized as “less than lower secondary”, “upper secondary or vocational training”, or “tertiary”. Marital status was defined as married if the participant was currently married and living with a spouse, and unmarried if the participant was currently separated, divorced from a spouse, widowed, or never married. The permanent address was defined either as urban or rural. Insurance and financial support were classified as either “Yes” or “No.”

#### Behavioral factors

Behavioral factors included the history of alcohol consumption, smoking, the number of cigarettes smoked each day, social activities, poor sleep quality, and nighttime sleep duration. History of alcohol consumption, smoking, and social activities was classified as either “Yes” or “No”. Poor sleep quality was assessed according to the response “my sleep was restless”, and divided into four groups according to the amount of time this statement was true during a week. Total nighttime sleep duration data was obtained from the question ‘‘During the past month, how many hours of actual sleep did you get at night (average hours for one night)?’’.

#### Health status

According to previous research and our professional knowledge [21–25], the factors selected as potentially predictive for frailty were a history of chronic disease (hypertension, dyslipidemia, cancer, heart disease, chronic lung disease, stroke, mental disease, arthritis or rheumatism, liver disease, kidney disease, digestive disease, or asthma), waist circumference, grip strength, self-perceived health status, ADL score, medication, vision, hearing, pain, and cognitive function.

Chronic disease and pain were based on self-reported diagnoses and defined as “Yes” or “No”. Self-perceived health status, vision, and hearing were categorized as “good”, “fair”, and “poor”. Activities of daily living were measured using The Katz Index of Independence in Activities of Daily Living (Katz ADL) [26], and six items were included in the CHARLS questionnaire: feeding, dressing, transferring, going to the toilet, bathing, and continence; 1 point was assigned for “No, I don’t have any difficulty” and “I have difficulty but can still do it”, and 0 points were assigned for “Yes, I have difficulty and need help” and “I cannot do it”; thus, total Katz ADL score indicates the degree of dependency, with lower scores indicating a higher level of dependency.

Cognitive functions include visuospatial skills, memory, orientation and attention. Visuospatial skills were assessed by redrawing a picture of two overlapped pentagons; those who redrew the picture correctly scored one point, while those who failed scored zero points. Memory was measured as the mean score for immediate and delayed recall of ten Chinese words; one point was given for each word correctly recalled. Orientation and attention were measured by the Telephone Interview of Cognitive Status (TICS-10), which calculates a score based on answers to questions regarding the year, month, day, day of the week, season, and serial subtraction of 7 from 100 (up to five times), with one point for each correct answer, and a total score of 0–10. The sum total of the above dimensions was the total cognitive function score, and ranged from zero to 21, with higher scores representing better cognitive function [27].

#### Mental health factors

Mental health factors included depression and life satisfaction. Depression was assessed in the questionnaire using the Center for Epidemiologic Studies Depression Scale (CES-D) [28], which is widely used as a measure of mental health status and has a total score for 10 items of 30 points, a score of 10 points or more are defined as depression. Life satisfaction was categorized as “good”, “fair” and “poor”.

#### Statistical methods

Data from the CHARLS database for 2013 and 2015 were selected for analysis in this study. Measures are expressed as median and interquartile range, and comparisons between groups were analyzed using the rank sum test. Categorical variables are expressed as percentages, and comparisons between groups were analyzed using the χ2 test or Fisher’s exact test. Data were randomly divided into training (*n* = 1005) and validation (*n* = 431) sets, according to a ratio of 7:3 [29].

A nomogram was used to illustrate the risk of frailty in individuals with diabetes, and the least absolute shrinkage and selection operator (LASSO) regression analysis was used to develop and validate the model. First, training set data were analyzed by LASSO regression [30, 31] to select predictors of frailty in people with diabetes. Then, tenfold cross-validation was applied to confirm the appropriate tuning parameters (λ) for LASSO regression analysis and the most significant features were screened with the LASSO algorithm. Finally, the selected predictors were included in multifactor logistic regression analysis, and those with *P* values < 0.05 were included in the nomogram model. Maximum missing values for all variables extracted did not exceed 20%, and multiple imputation was used to handle missing data [32].

Discrimination, accuracy, and clinical validity were used to validate the prediction model. In this study, the area under the receiver operating characteristic (ROC) curve (AUC) was used to determine the discrimination ability of the model. Calibration curves were used to determine the degree of agreement between predicted probabilities and observed outcomes. Decision curve analysis (DCA) was used to assess clinical validity.

R software (version 4.1.0) was used for all analyses in this study. All tests were two-tailed, and *P* < 0.05 was considered statistically significant.

## Results

### Participant characteristics

In total 1436 people with diabetes were included in this study. The demographic and clinical characteristics of participants are listed in Table 1. There were 586 male patients (40.8%), 850 female patients (59.2%), and 8.3% of patients were ≥ 75 years old. The internal validation cohort consisted of 431 patients, an additional file shows this in more detail (see Additional file).

**Table 1:** Baseline characteristics of the study population

Variable	Total	Non-frail	Frail	P
	*n* = 1436	*n* = 1291	*n* = 145	
ADL score	6.00 [5.00, 6.00]	6.00 [6.00, 6.00]	4.00 [3.00, 6.00]	< 0.001
Cognitive function	11.00 [7.50, 13.50]	11.50 [8.00, 13.50]	7.00 [4.00, 11.00]	< 0.001
Grip strength (kg)	28.65 [22.00, 36.00]	29.50 [23.00, 37.00]	21.50 [16.00, 26.90]	< 0.001
Waistline (cm)	91.80 [85.00, 99.00]	92.00 [85.40, 99.00]	88.70 [80.00, 95.00]	< 0.001
Nighttime sleep duration (h)	6.00 [5.00, 8.00]	6.00 [5.00, 8.00]	6.00 [4.00, 7.00]	< 0.001
Smoking per day	0.00 [0.00, 0.00]	0.00 [0.00, 0.00]	0.00 [0.00, 0.00]	0.149
Medications	2.00 [1.00, 3.00]	2.00 [1.00, 3.00]	2.00 [1.00, 3.00]	< 0.001
Age, years (%)				< 0.001
< 55	315 (21.9)	297 (23.0)	18 (12.4)	
55–64	613 (42.7)	561 (43.4)	52 (35.9)	
65–74	389 (27.1)	338 (26.2)	51 (35.2)	
≥ 75	119 (8.3)	95 (7.4)	24 (16.5)	
Gender (%)				< 0.001
Male	586 (40.8)	553 (42.8)	33(22.8)	
Female	850 (59.2)	738 (57.2)	112 (77.2)	
Education (%)				0.024
Less than lower secondary	1243 (86.6)	1107 (85.7)	136 (93.8)	
Upper secondary or vocational training	154 (10.7)	146 (11.3)	8 (5.5)	
Tertiary	39 (2.7)	38 (3.0)	1 (0.7)	
Marital status (%)				0.003
Married	1239 (86.4)	1126 (87.2)	113 (77.9)	
Unmarried	197 (13.6)	165 (12.8)	32 (22.1)	
Permanent address (%)				0.036
Urban	718 (50.0)	658 (51.0)	60 (41.4)	
Rural	718 (50.0)	633 (49.0)	85 (58.6)	
Self-perceived health status (%)				< 0.001
Good	190 (13.2)	183 (14.2)	7 (4.8)	
Fair	721 (50.2)	680 (52.7)	41 (28.3)	
Poor	525 (36.6)	428 (33.1)	97 (66.9)	
Hypertension (%)	787 (54.8)	693 (53.7)	94 (64.8)	0.014
Cancer (%)	33 (2.3)	31 (2.4)	2(1.4)	0.627
Chronic lung disease (%)	207 (14.4)	175 (13.6)	32 (22.1)	0.008
Heart disease (%)	428 (29.8)	372 (28.8)	56 (38.6)	0.019
Stroke (%)	106 (7.4)	92 (7.1)	14 (9.7)	0.349
Mental disease (%)	33 (2.3)	27 (2.1)	6 (4.1)	0.205
Arthritis or rheumatism (%)	607 (42.3)	513 (39.7)	94 (64.8)	< 0.001
Dyslipidemia (%)	611 (42.5)	549 (42.5)	62 (42.8)	1
Liver disease (%)	117 (8.1)	107 (8.3)	10 (6.9)	0.674
Kidney disease (%)	182 (12.7)	144 (11.2)	38 (26.2)	< 0.001
Digestive disease (%)	423 (29.5)	364 (28.2)	59 (40.7)	0.002
Asthma (%)	102 (7.1)	82 (6.4)	20 (13.8)	0.002
Alcohol consumption (%)	378 (26.3)	359 (27.8)	19 (13.1)	< 0.001
Smoking (%)	542 (37.7)	508 (39.3)	34 (23.4)	< 0.001
Insurance (%)	1367 (95.2)	1232 (95.4)	135 (93.1)	0.3
Social activities (%)	826 (57.5)	779 (60.3)	47 (32.4)	< 0.001
Financial support (%)	1089 (75.8)	970 (75.1)	119 (82.1)	0.081
Poor sleep quality (%)				< 0.001
Rarely or none of the time	679 (47.3)	639 (49.5)	40 (27.6)	
Some or a little of the time	219 (15.2)	193 (14.9)	26 (17.9)	
Occasionally or a moderate amount of the time	217 (15.1)	191 (14.8)	26 (17.9)	
Most or all of the time	321 (22.4)	268 (20.8)	53 (36.6)	
Depression (%)	1089 (75.8)	970 (75.1)	119 (82.1)	< 0.001
Life satisfaction (%)				< 0.001
Good	475 (33.1)	439 (34.0)	36 (24.8)	
Fair	787 (54.8)	715 (55.4)	72 (49.7)	
Poor	174 (12.1)	137 (10.6)	37 (25.5)	
Vision (%)				0.001
Good	253 (17.6)	235 (18.2)	18 (12.4)	
Fair	636 (44.3)	585 (45.3)	51 (35.2)	
Poor	547 (38.1)	471 (36.5)	76 (52.4)	
Hearing (%)				< 0.001
Good	512 (35.7)	478 (37.0)	34 (23.4)	
Fair	711 (49.5)	642 (49.7)	69 (47.6)	
Poor	213 (14.8)	171 (13.3)	42 (29.0)	
Pain (%)	536 (37.3)	438 (33.9)	98 (67.6)	< 0.001

### Prevalence of frailty and related variables

The prevalence of frailty was 10.1% (145/1436). Several factors, including ADL, cognitive function, grip strength, and waistline differed significantly (*P* < 0.05) between patients with and without frailty. Of patients with diabetes, 1005 (70%) and 431 (30%) were randomly assigned to the training and validation sets, respectively. Comparisons between the training and validation sets are presented in the additional file, and no significant differences were detected between the two groups (*P* > 0.05).

### LASSO and logistic regression of patients with diabetes

**Fig. 1:**
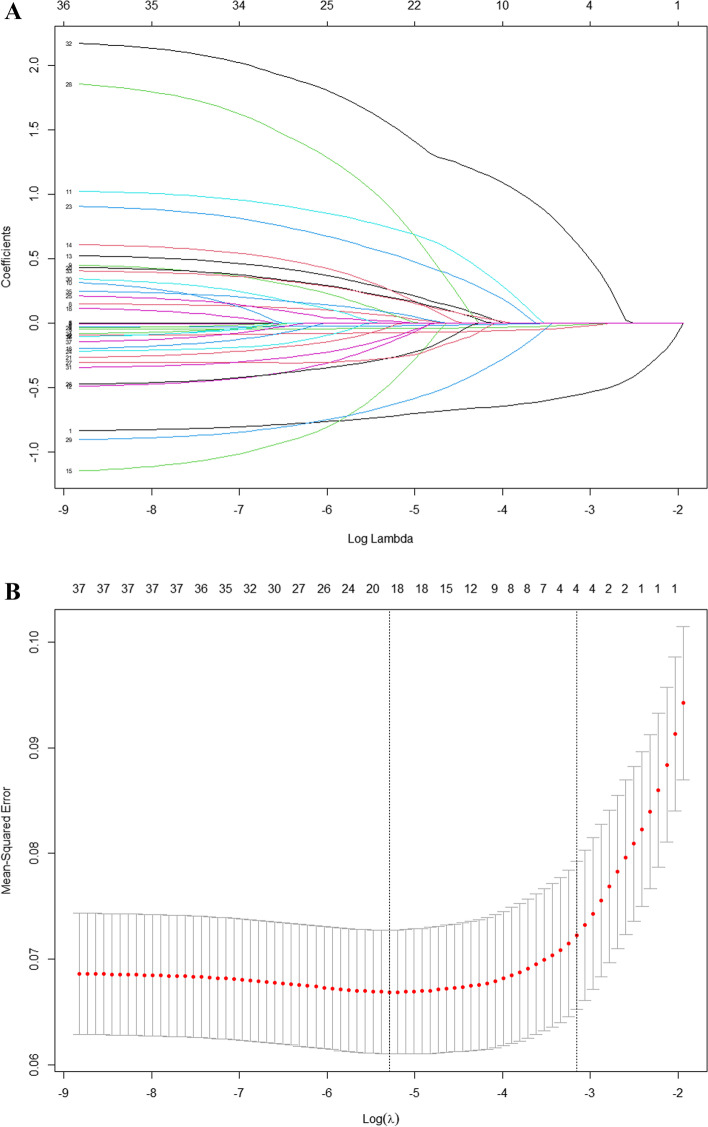
Demographic and clinical feature selection using the LASSO regression model. **A** According to the logarithmic (lambda) sequence, a coefficient profile was generated, and non-zero coefficients were produced by the optimal lambda. **B** The optimal parameter (lambda) in the LASSO model was selected via tenfold cross-validation using minimum criteria. The partial likelihood deviation (binomial deviation) curve relative to log (lambda) was plotted. A virtual vertical line at the optimal value was drawn using one SE of minimum criterion (the 1-SE criterion)

In the LASSO regression model, this study has chosen non-zero coefficients as potential predictors of frailty (Fig. 1A and B). And then, we further used the ‘rms’ package in ‘R’ software to incorporate these potential factors related to frailty into the multivariate logistic regression model. Ultimately, marital status (*P* = 0.003), ADL (*P* < 0.001), waistline (*P* = 0.017), cognitive function (*P* = 0.008), grip strength (*P* < 0.001), social activity (*P* = 0.003), and depression (*P* < 0.001) were associated with the development of frailty in patients with diabetes (Table 2).

**Table 2:** The prediction model with multivariate logistic regression

Variable	Multivariate analysis OR (95%CI)	P
ADL score	0.44(0.37–0.53)	< 0.001
Waist circumference	0.98(0.96–1.00)	0.017
Cognitive function	0.92(0.86–0.98)	0.008
Grip strength	0.95(0.92–0.97)	< 0.001
Marital status		
Married	Reference	
Unmarried	2.67(1.40–5.02)	0.003
Social activities		
Yes	0.44(0.25–0.75)	0.003
No	Reference	
Depression		
Yes	5.40(3.14–9.57)	< 0.001
No	Reference	

### Predictive model development

**Fig. 2:**
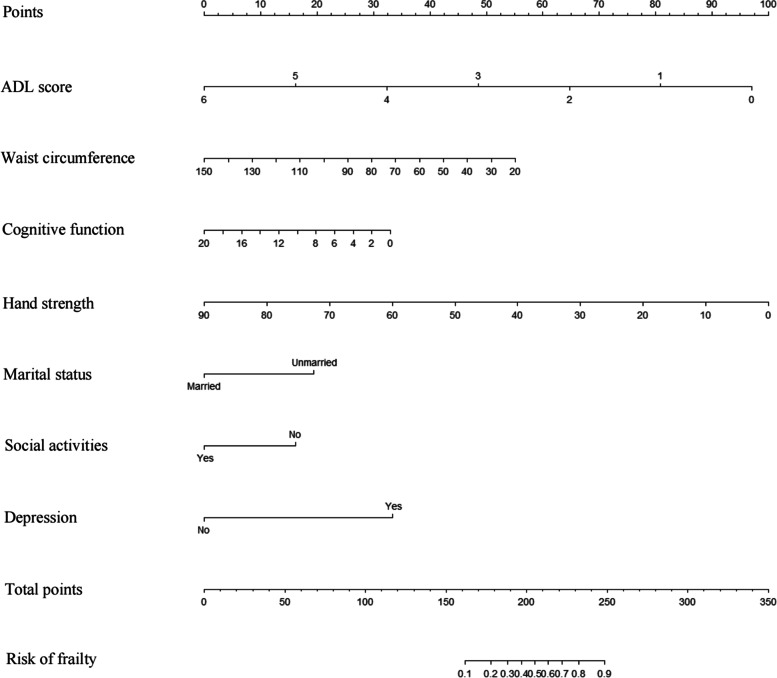
Nomogram

LASSO regression analysis was used to screen the variables for the best predictors of the model based on a 10-fold cross-validation. Multivariate logistic regression was conducted to establish a predictive model. The variance inflation factor (VIF) test was performed, and VIF values for all variables were < 4. There was no covariance and the model fit was good. The prediction model was composed of variables with P values that were less than 0.05 in the multivariate logistic regression. These variables included marital status, ADL, waistline, cognitive function, grip strength, social activity, and depression as predictors. The predictive model was presented using a nomogram, which can be used to quantitatively predict the risk of frailty in patients with diabetes (Fig. 2).

### Predictive model validation

#### Discrimination

**Fig. 3:**
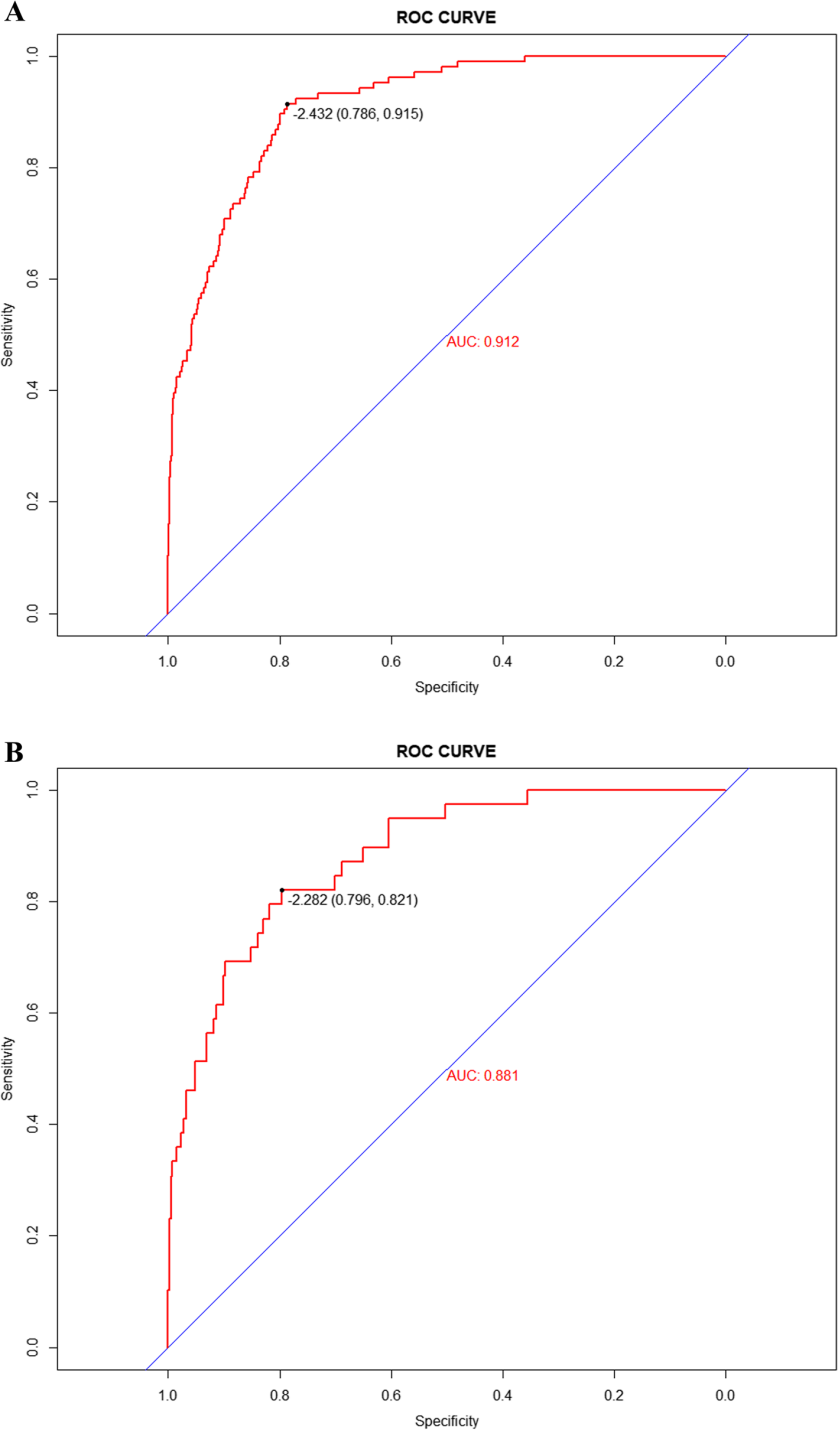
**A** Nomogram ROC curves generated from the training dataset. **B** Nomogram ROC curves generated using the validation dataset

AUC values were calculated to assess the discrimination of the predictive model by examining the occurrence of frailty in patients with diabetes in the training and validation sets. As shown in Fig. 3A and B, the predictive model yielded an AUC value of 0.912 (95% CI = 0.887–0.937), with a specificity of 0.786 and sensitivity of 0.915, in the training set, and AUC = 0.881 (95% CI = 0.829–0.934), with a specificity of 0.796 and sensitivity of 0.821, in the validation set. These data indicate that the nomogram has good discriminatory ability and predictive value, and can correctly identify frail and non-frail patients.

### Calibration of the predictive model

**Fig. 4:**
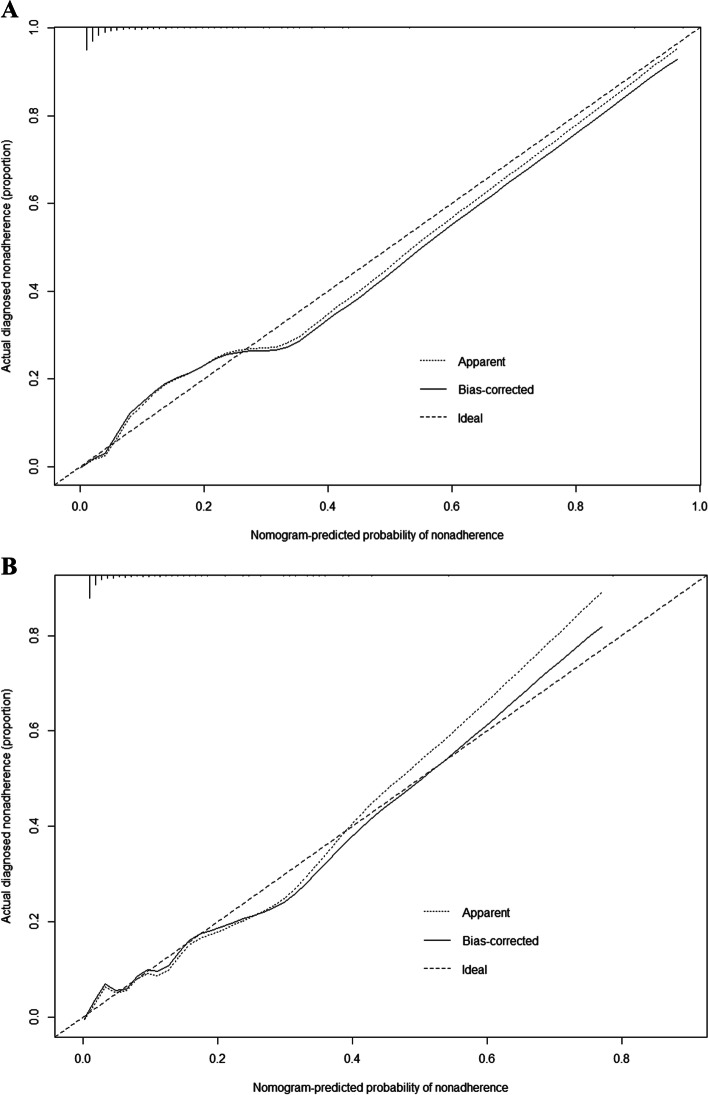
**A** Calibration plot for the training dataset. **B** Calibration plot for the validation dataset

The nomogram was evaluated using a calibration plot and the Hosmer–Lemeshow goodness-of-fit test (*P* > 0.05 indicates that the model exhibits a very good degree of fit). The test results showed that the model had a very good fit for both the training (χ2 = 4.3518, df = 8, *p* = 0.8241) and validation (χ2 = 6.3492, df = 8, *p* = 0.6082) sets. Calibration plots for the training and validation sets, based on the multifactorial logistic regression model, are shown in Fig. 4A and B. Calibration curves for the nomogram showed high uniformity between the predicted and actual probabilities of frailty in the training (Fig. 4A) and validation (Fig. 4B) sets.

### Evaluation of clinical validity

**Fig. 5:**
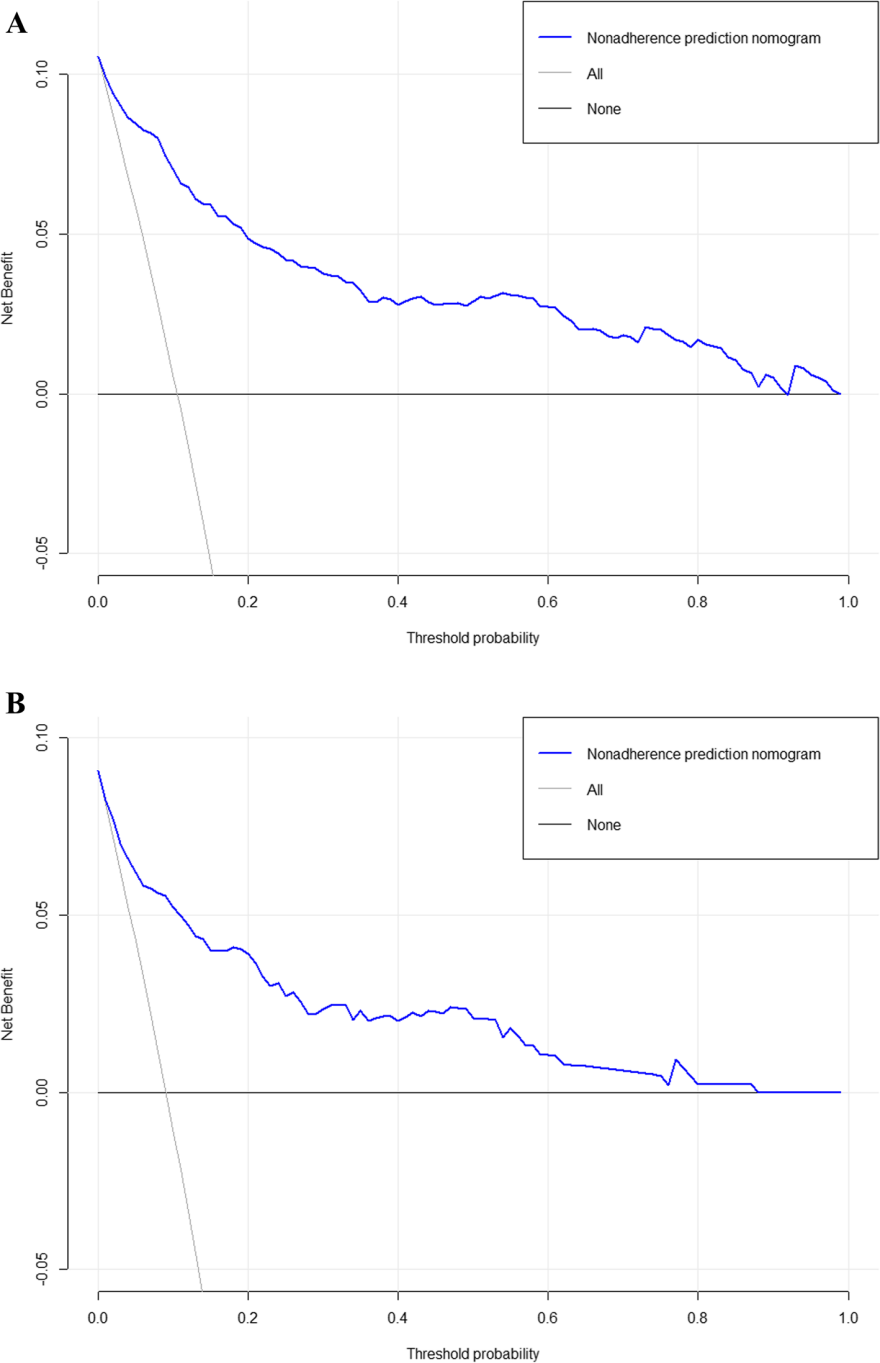
**A** DCA curves for the training dataset. **B** DCA curves for the validation dataset

The clinical validity of the model was evaluated using the DCA method, and the results are shown in Fig. 5A and B. From the decision curves, the net benefits of the predictive model for the internal validation set were significantly higher than those of the two extreme cases, indicating that the nomogram model had the superior net benefit and predictive accuracy.

## Discussion

The occurrence of frailty in patients with diabetes in the present study was 10.1%, which is consistent with previous reports of the prevalence of frailty, which range from 3.9% to 17.1% [33, 34]. Frailty is closely related to the occurrence of falls, fractures, and death [35]. Hence, the identification of individuals at high risk is important to prevent frailty and associated adverse outcomes, especially in the early stages of diabetes.

The pathogenesis of frailty is complex and associated with multiple factors.

This study revealed that marital status was a predictor of frailty in patients with diabetes. The results showed that unmarried individuals with diabetes (including those who were divorced, widowed, and never married) were more likely to develop frailty than those who were married, which is consistent with previous findings [36, 37]. Unmarried people usually live alone and lack psychological and social support in the face of stressful events, then they are more likely to experience a sense of social isolation and loneliness, which further aggravate the development of frailty [16]. Married people are more accessible to family or social support to help with their diabetes self-management, then they are often at a lower risk of frailty. Therefore, unmarried patients deserve more attention from professionals to protect them from frailty and help them to maintain a high quality of life.

Our predictive model showed that low ADL scores were also associated with frailty. Diabetics with impaired ADL were more likely to develop frailty. A previous cross-sectional study also proved the relationship between functional incapacity and frailty and further confirmed that ADL score is a predictor of frailty [34]. ADL reflects an individual’s self-care ability. Patients with impaired ADL generally also experience reduced self-care ability, which may further influence their eating habits and can result in malnutrition. Additionally, reduced physical function leads to reduced activity levels, which may result in decreased muscle strength and bone density, leaving patients susceptible to sarcopenia and osteoporosis, which can further lead to a high risk of frailty [38]. Therefore, the inclusion of ADL in the routine assessment of patients with diabetes could help healthcare providers to conduct risk stratification and to develop interventions with positive effects in reducing frailty and other adverse health outcomes.

This study also found that hand grip strength and waist circumference were independent predictors of frailty. Patients with diabetes with low maximal hand grip strength in the main hand were more likely to develop frailty. Hand grip strength usually reflects muscle force level, which reflects physical function and physiological reserve capacity to some extent [39]. A decrease in hand grip strength represents a decrease in muscle mass and density, which will lead to loss of muscle force and motor coordination, thereby accelerating the onset of frailty [40]. Additionally, an important pathophysiological feature of frailty is skeletal muscle loss [41, 42], which is an important underlying physiological mechanism that supports our findings. Similarly, we also found that a smaller waist circumference was associated with a higher risk of developing frailty. This differs from the findings of previous studies that focus on healthy populations, indicating that abdominal obesity, presenting as a large waist circumference, is a risk factor for frailty [43–46]. Diabetes mellitus is a chronic wasting disease that causes weight loss or emaciation that can result in reduced waist circumference, which predisposes to malnutrition and sarcopenia [47]. Sarcopenia and chronic malnutrition are strongly associated with the development of frailty and both increase the risk of its development [48]. Additionally, weight loss is one of the criteria for frailty. Our data suggest that early nutritional intervention and muscle exercises should be provided for those at risk of malnutrition or reduced muscle strength to reduce the risk of frailty.

Moreover, this study found that frailty is closely associated with cognitive function. The cognitive function of the frail group was significantly lower than that of the non-frail group in the present study. Lower cognitive function was associated with a higher risk of frailty. This is consistent with the results of a previous study, indicating frailty was associated with subjective cognitive decline [49]. The relationship between cognitive function and frailty could be explained by pathogenic mechanisms common to them both, such as chronic inflammation and oxidative stress [50]. The common pathogenic mechanisms allow them to interact and contribute to one another. Besides, diabetes, as an important risk factor for Alzheimer’s disease, can accelerate cognitive decline [51]. The cognitive decline will reduce patients’ self-management ability and compliance with diabetes treatment, which further aggravates the disease progression and contribute to a higher risk of frailty. Thus, the cognitive function should be taken seriously in patients with diabetes and those with cognitive decline should take cognitive training as early as possible to slow the decline of cognitive function and help to prevent frailty.

The present study also found that depression and social activity were associated with frailty in patients with diabetes, which is supported by previous studies [52]. Some studies have proved that depression and frailty have the same pathophysiologic mechanisms [53]. Additionally, depressive symptoms can adversely affect psychological conditions and aggravate the onset of frailty by reducing social activity. The prevalence of depression in patients with diabetes is as high as 15%, which is approximately twice as high as in non-diabetics [54]. Our study indicated that social activity is a protective factor against frailty. Patients who were socially active had a lower risk of frailty than those who never or rarely socialized. These findings are supported by previous studies, showing that social activities can reduce loneliness and social isolation, and are associated with a reduced prevalence of frailty [55, 56]. Furthermore, social activities can enable patients to acquire disease knowledge and management skills, build confidence to overcome the disease, improve self-management ability and self-efficacy, and prevent the occurrence of diabetes complications [57]. It is helpful in slowing down the process of frailty development. Hence, more experience-sharing and various forms of social activities should be organized for patients with diabetes. Healthcare professionals should pay attention to the mental health of these patients and be alert to negative emotions to prevent depression and the development of frailty.

The nomogram is a commonly used prediction model used in research in many clinical fields. Nomograms are quantitative analysis diagrams that represent the functional relationship between variables using planar coordinates connected by disjointed line segments, which can be applied to predict the probability of a clinical outcome event by adding up the scores of each predictor to obtain a total score [58]. No nomograms for predicting frailty in patients with diabetes based on population-based data have been reported previously. In this study, we found that marital status, ADL, waist circumference, cognitive function, grip strength, social activity, and depression were the main factors predicting frailty in patients with diabetes. Our predictive model, constructed based on these seven factors influencing the development of frailty, demonstrated good discrimination, calibration, and clinical validity, indicating that the prediction model is valuable for the effective identification of individuals with diabetes at high risk of developing frailty. The nomogram can specifically quantify the hazard ratio in the form of a score, the probability of a patient developing a certain outcome can be obtained by simple calculation, and it can provide personalized risk assessment for each individual, which is highly relevant and accurate. Therefore, the establishment of a predictive model for frailty in patients with diabetes is a novel achievement of this study. As an efficient and accurate assessment tool, our predictive model can assist medical practitioners in screening for individuals with diabetes at high risk of developing frailty and provides a theoretical basis and entry point for the development of early prevention and intervention measures. The predictive model demonstrated good clinical applicability and it was helpful in screening patients at high risk for frailty.

There are some limitations in the present study. First, some potential predictors, including diet habits, hypoglycemia, and some diabetes complications, were not provided in the CHARLS database. Second, the nomogram was developed based on data from China, and whether the results of this study can be extended to other regions and countries requires further verification using data from external cohorts. Third, this was a retrospective study and the patients with diabetes were not followed up, hence data from more patients who have undergone long-term follow-up should be analyzed to improve the current nomogram model.

## Conclusion

This study established and verified a nomogram model that can predict frailty in patients with diabetes. Our nomogram model, which combines marital status, ADL, waist circumference, cognitive function, grip strength, social activity, and depression, was verified internally as a useful tool for risk assessment. The developed predictive model will be valuable in screening patients with diabetes at high risk for frailty.

## Supplementary Information

**Additional file 1.** Comparison between variables in the training and validation datasets.

## Data Availability

The datasets generated during and/or analyzed during the current study are available in the CHARLS repository, http://charls.pku.edu.cn.

## Abbreviations

ROC: Receiver operating characteristic
AUC: Area under the curve
DCA: Decision curve analysis
ADL: Activities of daily living
OR: Odds ratio
CI: Confidence interval
