# Supplementary material for: Development and validation of a risk prediction model for frailty in patients with diabetes
Source: BMC Geriatr. 2023 Mar 27;23:172. doi: 10.1186/s12877-023-03823-3 (PMC10045211; doi:10.1186/s12877-023-03823-3)
Supplement: Supplementary file 1 — Additional file 1. Comparison between variables in the training and validation datasets. [file 12877_2023_3823_MOESM1_ESM.docx]

**Additional file**

Title of data: Comparison between variables in the training and validation datasets

**Comparison between variables in the training and validation datasets**

| Variables | Total | Training set | Validation set | P |
| --- | --- | --- | --- | --- |
|  | 1436 | n = 1005 | n = 431 |  |
| ADL score | 6.00 [5.00, 6.00] | 6.00 [5.00, 6.00] | 6.00 [6.00, 6.00] | 0.131 |
| Cognitive function | 11.00 [7.50, 13.50] | 11.00 [7.50, 13.50] | 11.00 [8.00, 13.50] | 0.924 |
| Grip strength (kg) | 28.65 [22.00, 36.00] | 28.00 [22.00, 36.00] | 30.00 [22.00, 37.25] | 0.128 |
| Waistline (cm) | 91.80 [85.00, 99.00] | 92.00 [84.90, 99.00] | 91.50 [85.00, 98.00] | 0.722 |
| Nighttime sleep duration (h) | 6.00 [5.00, 8.00] | 6.00 [5.00, 7.50] | 6.00 [5.00, 8.00] | 0.694 |
| Smoking per day | 0.00 [0.00, 0.00] | 0.00 [0.00, 0.00] | 0.00 [0.00, 0.00] | 0.336 |
| Medications | 2.00 [1.00, 3.00] | 2.00 [1.00, 3.00] | 2.00 [1.00, 3.00] | 0.48 |
| Age, years (%) |  |  |  | 0.674 |
| ＜55 | 315 (21.9) | 214 (21.3) | 101 (23.4) |  |
| 55–64 | 613 (42.7) | 430 (42.8) | 183 (42.5) |  |
| 65–74 | 389 (27.1) | 273 (27.2) | 116 (26.9) |  |
| ≥ 75 | 119 (8.3) | 88 (8.7) | 31 (7.2) |  |
| Gender (%) |  |  |  | 0.438 |
| Male | 586 (40.8) | 403 (40.1) | 183 (42.5) |  |
| Female | 850 (59.2) | 602 (59.9) | 248 (57.5) |  |
| Education (%) |  |  |  | 0.5 |
| Less than lower secondary | 1243 (86.6) | 872 (86.8) | 371 (86.1) |  |
| Upper secondary or vocational training | 154 (10.7) | 109 (10.8) | 45 (10.4) |  |
| Tertiary | 39 (2.7) | 24 (2.4) | 15 (3.5) |  |
| Marital status (%) |  |  |  | 0.544 |
| Married | 1239 (86.3) | 863 (85.9) | 376 (87.2) |  |
| Unmarried | 197 (13.7) | 142 (14.1) | 55 (12.8) |  |
| Permanent address (%) |  |  |  | 0.49 |
| Urban | 718 (50.0) | 496 (49.4) | 222 (51.5) |  |
| Rural | 718 (50.0) | 509 (50.6) | 209 (48.5) |  |
| Self-perceived health status (%) |  |  |  | 0.389 |
| Good | 190 (13.2) | 125 (12.4) | 65 (15.1) |  |
| Fair | 721 (50.2) | 511 (50.9) | 210 (48.7) |  |
| Poor | 525 (36.6) | 369 (36.7) | 156 (36.2) |  |
| Hypertension (%) | 787 (54.8) | 562 (55.9) | 225 (52.2) | 0.215 |
| Cancer (%) | 33 (2.3) | 25 (2.5) | 8 (1.9) | 0.589 |
| Chronic lung disease (%) | 207 (14.4) | 143 (14.2) | 64 (14.8) | 0.822 |
| Heart disease (%) | 428 (29.8) | 311 (30.9) | 117 (27.1) | 0.168 |
| Stroke (%) | 106 (7.4) | 74 (7.4) | 32 (7.4) | 1 |
| Mental disease (%) | 33 (2.3) | 17 (1.7) | 16 (3.7) | 0.032 |
| Arthritis or rheumatism (%) | 607 (42.3) | 428 (42.6) | 179 (41.5) | 0.754 |
| Dyslipidemia (%) | 611 (42.5) | 433 (43.1) | 178 (41.3) | 0.569 |
| Liver disease (%) | 117(8.1) | 81 (8.1) | 36 (8.4) | 0.936 |
| Kidney disease (%) | 182 (12.7) | 126 (12.5) | 56 (13.0) | 0.88 |
| Digestive disease (%) | 423 (29.5) | 290 (28.9) | 133 (30.9) | 0.484 |
| Asthma (%) | 102 (7.1) | 66 (6.6) | 36 (8.4) | 0.273 |
| Alcohol consumption (%) | 378 (26.3) | 264 (26.3) | 114 (26.5) | 0.995 |
| Smoking (%) | 542 (37.7) | 373 (37.1) | 169 (39.2) | 0.489 |
| Insurance (%) | 1367 (95.2) | 964 (95.9) | 403 (93.5) | 0.068 |
| Social activities (%) | 826 (57.5) | 566 (56.3) | 260 (60.3) | 0.177 |
| Financial support (%) | 1089 (75.8) | 768 (76.4) | 321 (74.5) | 0.472 |
| Poor sleep quality (%) |  |  |  | 0.07 |
| Rarely or none of the time | 679 (47.3) | 470 (46.8) | 209 (48.5) |  |
| Some or a little of the time | 219 (15.3) | 147 (14.6) | 72 (16.7) |  |
| Occasionally or a moderate amount of the time | 217 (15.1) | 168 (16.7) | 49 (11.4) |  |
| Most or all of the time | 321 (22.4) | 220 (21.9) | 101 (23.4) |  |
| Depression (%) | 499 (34.7) | 355 (35.3) | 144 (33.4) | 0.524 |
| Life satisfaction (%) |  |  |  | 0.761 |
| Good | 475 (33.1) | 327 (32.5) | 148 (34.3) |  |
| Fair | 787 (54.8) | 557 (55.4) | 230 (53.4) |  |
| Poor | 174 (12.1) | 121 (12.0) | 53 (12.3) |  |
| Vision (%) |  |  |  | 0.669 |
| Good | 253 (17.6) | 183 (18.2) | 70 (16.2) |  |
| Fair | 636 (44.3) | 442 (44.0) | 194 (45.0) |  |
| Poor | 547 (38.1) | 380 (37.8) | 167 (38.7) |  |
| Hearing (%) |  |  |  | 0.423 |
| Good | 512 (35.7) | 354 (35.2) | 158 (36.7) |  |
| Fair | 711 (49.5) | 508 (50.5) | 203 (47.1) |  |
| Poor | 213 (14.8) | 143 (14.2) | 70 (16.2) |  |
| Pain (%) | 536 (37.3) | 379 (37.7) | 157 (36.4) | 0.668 |

Description of data: the additional file groups participants according to the training and validation sets, and perform statistical analysis of the basic characteristics of the two groups, with the results presented as a table.
